# Association between pregnancy-related changes in serum creatinine and preeclampsia diagnosis

**DOI:** 10.1007/s00404-024-07685-x

**Published:** 2024-08-16

**Authors:** Lynsa M. Nguyen, Shelby A. Crants, Christin M. Giordano, Lisa C. Zuckerwise, Matthew R. Grace

**Affiliations:** 1 Department of Obstetrics and Gynecology, Division of Maternal Fetal Medicine, Vanderbilt University Medical Center, Nashville, TN USA; 2 Department of Obstetrics and Gynecology, Brigham and Women’s Hospital, 75 Francis Street CWN-3, Boston, MA 02115 USA; 3 Department of Internal Medicine, Division of Nephrology and Hypertension, Vanderbilt University Medical Center, Nashville, TN USA

**Keywords:** Preeclampsia, Creatinine, Pregnancy, Renal function

## Abstract

**Background:**

Preeclampsia is a leading cause of maternal and neonatal morbidity and mortality, affecting 2–8% of all pregnancies. Typically, the increased glomerular filtration rate of pregnancy results in a decrease in serum creatinine. It is unknown if women without the expected decrease in serum creatinine during pregnancy are more likely to be diagnosed with preeclampsia.

**Objective:**

We sought to determine if the absence of a pregnancy-related decrease in serum creatinine was associated with the development of preeclampsia in patients deemed to be at high risk for developing preeclampsia. We hypothesized that the absence of the expected decrease in serum creatinine may be a marker of impaired renal function and therefore may be associated with increased risk of preeclampsia in this cohort.

**Study design:**

We conducted a retrospective cohort study of deliveries between November 2, 2017 and June 30, 2020 at a single institution. Pregnancies were included if a baseline serum creatinine (measured between one year prior to conception through 6 weeks gestation), and another serum creatinine value prior to 20 weeks of gestation were measured. Decrease in serum creatinine was defined as any decrease (at least 0.01 mg/dL) from baseline. The primary outcome was diagnosis of preeclampsia. Exclusion criteria included fetal anomalies, fetal demise, multiple gestation, or delivery prior to 20 weeks. Bivariable analyses were performed using Chi-square, ANOVA, and Student’s t test. Logistic regression was used to determine odds of developing preeclampsia controlling for confounders.

**Results:**

We identified 392 pregnancies that met inclusion criteria. Preeclampsia was diagnosed in 56 (14.3%) pregnancies. Patients diagnosed with preeclampsia were more likely to have a history of preeclampsia in a prior pregnancy, chronic hypertension (HTN), and diabetes. They were also more likely to have aspirin prescribed in the current pregnancy. Prevalence of advanced maternal age, multiparity, obesity, smoking, history of autoimmune disease, history of CKD, gestational HTN, or multiple pregnancy were not significantly different between patients with and without a diagnosis of preeclampsia. After controlling for confounders, a decrease in serum creatinine from baseline was not significantly associated with a diagnosis of preeclampsia (OR 0.76, CI 0.32–1.78).

**Conclusion:**

After controlling for risk factors associated with preeclampsia, a decrease in serum creatinine from baseline was not significantly associated with a diagnosis of preeclampsia in this high-risk cohort.

## What does this study add to the clinical work

**Table:** 

This is the first study to our knowledge that investigates an absence of a physiologic decrease in creatinine in early pregnancy as a potential risk factor for preeclampsia. Though this study did not find a significant relationship between serum creatinine decrease and preeclampsia risk, this study does serve as a reminder that, given the benefits of low-dose aspirin for patients at risk of developing preeclampsia, additional risk factors should continue to be explored and identified.	

## Introduction

Preeclampsia is a hypertensive disorder of pregnancy and a leading cause of maternal and neonatal morbidity and mortality in the United States [1]. Preeclampsia can lead to many acute maternal health complications, including increased risk of seizure, stroke, acute kidney injury, and disseminated intravascular coagulation [2]. Pregnancies complicated by preeclampsia are also associated with long-term maternal risk of early mortality due to development of various chronic diseases, including cardiovascular disease, stroke, and diabetes [2]. Preeclampsia is also associated with neonatal risks including low birthweight, bronchopulmonary dysplasia, and cerebral palsy [2]. Though the exact pathogenesis of preeclampsia is still unknown, numerous risk factors have been identified, such as history of preeclampsia, multifetal gestation, chronic hypertension, pre-gestational diabetes, autoimmune disorders, and renal disease [1].

Pregnancy is associated with a variety of physiologic changes, including modifications in the structure and function of the genitourinary system [3]. Alterations in the renin–angiotensin–aldosterone system and elevation in progesterone levels mediate a vasodilated state in pregnancy and changes in renal tubular function [3]. These changes, combined with the increase in plasma volume during pregnancy, result in an increase in glomerular filtration rate by approximately fifty percent [4]. This is reflected by an overall decrease in serum creatinine levels that can be seen as early as 6 weeks of gestation [4]. The precise regulation of hemodynamic changes of pregnancy is critical for a healthy pregnancy, and failure of glomerular hyperfiltration in pregnancy may be reflective of underlying maternal renal dysfunction, which is a known risk factor for preeclampsia. [5].

Preeclampsia causes vasospasm of dysfunctional endothelial cells leading to contraction of the intravascular space. Consequently, the physiologic increase in renal blood flow, increase in glomerular filtration, and decrease in serum creatinine that are associated with pregnancy may not occur normally in patients who develop preeclampsia [6]. A decrease in renal perfusion secondary to decreased intravascular volume and intrarenal vasospasm can lead to an increase in serum creatinine during pregnancy due to decreased creatinine clearance or acute renal injury [6]. Previous research in this area has also demonstrated that early trimester serum creatinine may be related to the interval between the onset of increased blood pressure and proteinuria in patients who develop preeclampsia [7]. The underlying pathophysiology of endothelial dysfunction is common between chronic kidney disease and preeclampsia, which may explain why those with chronic renal disease are predisposed to developing preeclampsia [9].

The objective of this study was to determine if the absence of the physiologic decrease in serum creatinine during pregnancy is associated with the development of preeclampsia. We hypothesized that patients with a decrease in their baseline serum creatinine, which would reflect a physiologic response to pregnancy, would be less likely to develop preeclampsia compared to patients who did not have a decrease in their serum creatinine.

## Methods

### Study design

This was a retrospective cohort study of deliveries at a single institution between November 2, 2017 and June 30, 2020. The study was approved by the Vanderbilt University Medical Center Institutional Review Board. Pregnant patients who delivered at Vanderbilt during this time were included if they had an available baseline serum creatinine and an additional serum creatinine measurement between 6 and 20 weeks of gestation. Baseline serum creatinine was defined as a serum creatinine measured between 1 year prior to conception through 6 weeks of gestation. A second serum creatinine measurement between 6 and 20 weeks was utilized for comparison, and a decrease in serum creatinine was defined as a decrease of at least 0.01 mg/dL from the baseline value. All serum creatinine measurements were obtained in the Vanderbilt University Medical Center laboratory using the kinetic alkaline picrate methodology.

Exclusion criteria included delivery prior to 20 weeks, fetal anomaly, fetal demise, end stage renal disease (ESRD), exposure to Lovenox during pregnancy, multiple gestation, and delivery at an outside institution.

### Assessment of outcomes of interest

The authors reviewed eligible charts and manually extracted pertinent lab values, patient characteristics, and diagnoses. The diagnosis of preeclampsia was made by the clinical team using criteria defined by the American College of Obstetrics & Gynecology [6]. We included patients with preeclampsia diagnosed antepartum, intrapartum, or postpartum. Other patient characteristics that were abstracted included maternal age at delivery, gestational age at delivery, race, gravidity/parity, pre-pregnancy body mass index (BMI), smoking status, history of preeclampsia in a prior pregnancy, use of aspirin, history of autoimmune disorder, gestational hypertension, chronic hypertension, chronic kidney disease, pre-gestational diabetes, and gestational diabetes.

### Statistical analysis

Bivariable analyses were performed using Chi-square, ANOVA, and Student’s *t* test. Multivariable logistic regression models were used to estimate odds ratios (ORs) and 95% *confidence intervals* (CI) for the primary outcome adjusted for potential confounders to determine the association between decrease in serum creatinine and preeclampsia diagnosis. Calculations were performed using STATA version 14.2 (StataCorp, College Station, Tx). A *p* value of < 0.05 was considered significant.

## Results

**Fig. 1:**
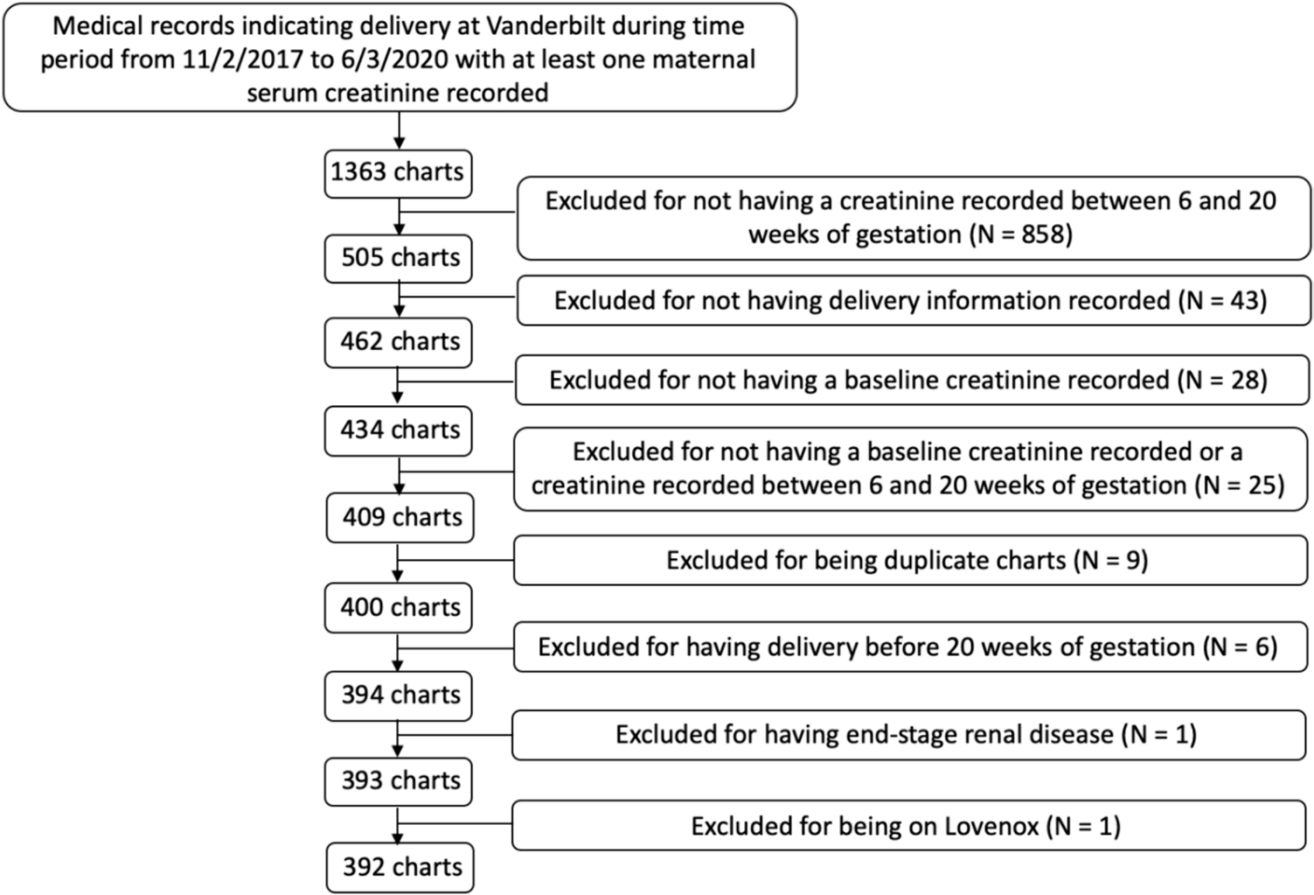
Flowchart for identification and selection of patients

Diagnostic codes for pregnancy were used to identify a total of 1363 medical records in which there was at least one serum creatinine recorded and a delivery occurring at Vanderbilt during our study time period. Of these medical records, a total of 392 deliveries were ultimately eligible for inclusion (Fig. 1).

Patient characteristics for our study population are shown in Table 1. There were no statistically significant differences in rates of advanced maternal age, race, multiparity, obesity, smoking status, autoimmune disorders, gestational diabetes, chronic kidney disease, gestational hypertension, or multiple gestation among those who developed preeclampsia compared to those who did not. In total, 56 (14.3%) of our 392 patients developed preeclampsia, 37 (66.1%) of whom had been prescribed aspirin during pregnancy. Patients who developed preeclampsia were significantly more likely to have a history of preeclampsia in a prior pregnancy (*p* = 0.001), chronic hypertension (*p* < 0.001), pre-gestational diabetes (*p* < 0.001), and aspirin use during pregnancy (*p* < 0.001). Aspirin was prescribed to 138 (35.4%) of our 392 patients, with 37 (26.8%) of these patients being diagnosed with preeclampsia, and 101 (73.2%) of these patients not being diagnosed with preeclampsia. A decrease in serum creatinine was noted in 189 (48.2%) of the 392 patients, 23 (41.1%) among patients with preeclampsia and 166 (49.4%) among patient without preeclampsia. This was not a statistically significant difference between these groups (*p* = 0.25) in unadjusted comparisons. After controlling for confounders, logistic regression models also did not demonstrate a significant association between a pregnancy-related decrease in serum creatinine and the development of preeclampsia in this cohort (OR 0.76, CI 0.32–1.78). A history of preeclampsia in prior pregnancy, chronic hypertension, and type 2 diabetes mellitus were each associated with increased odds of developing preeclampsia in this cohort (Table 2).

**Table 1:** Demographic data

	Study size (*N* = 392)	Diagnosis of preeclampsia		*p* value
Patient characteristics	Number of pregnancies (%)	No (*N* = 336)	Yes (*N* = 56)	
Average maternal age	392	29.7	29.9	0.8
Advanced maternal age	94 (24.0)	82 (24.4)	12 (21.4)	.63
Race				.39
African American	115 (29.3)	94 (28.0)	21 (37.5)	
Caucasian	237 (60.5)	204 (60.7)	33 (58.9)	
Asian	11 (2.8)	11 (3.3)	0 (0.0)	
Other	21 (5.4)	20 (6.0)	1 (1.8)	
Unknown	6 (1.5)	5 (1.5)	1 (1.8)	
Pacific Islander	2 (0.5)	2 (0.6)	0 (0.0)	
Average parity	392	1.3	1.2	0.5
Multiparity	259 (66.1)	227 (67.6)	32 (57.1)	.13
Obesity	197 (50.3)	163 (48.5)	34 (60.7)	.09
Smoking status				.88
Never smoked	262 (66.8)	223 (66.4)	39 (69.6)	
Smoked	67 (17.1)	59 (17.6)	8 (14.3)	
Other	63 (16.1)	54 (16.1)	9 (16.1)	
Autoimmune disorder	75 (19.1)	66 (19.6)	9 (16.1)	.53
History of preeclampsia	53 (13.6)	38 (11.3)	15 (27.3)	.001
Chronic hypertension	85 (21.8)	55 (16.5)	30 (53.6)	< .001
Diabetes mellitus				< .001
Type 1	28 (7.2)	17 (5.1)	11 (19.6)	
Type 2	24 (6.1)	16 (4.8)	8 (14.3)	
Gestational diabetes mellitus	29 (7.4)	27 (8.0)	2 (3.6)	.24
Chronic kidney disease	29 (7.4)	24 (7.1)	5 (8.9)	.64
Gestational hypertension	41 (11.2)	31 (9.9)	10 (18.5)	.06
Multiples	7 (1.8)	7 (2.1)	0 (0.0)	.28
Aspirin Use	138 (35.4)	101 (30.2)	37 (66.1)	< .001
Baseline serum creatinine	392	0.75	0.80	0.08
Early pregnancy serum creatinine (6–20 weeks of gestation)	392	0.65	0.70	0.10
≥0.01 mg/dL Decrease in Serum Creatinine	189 (48.2)	166 (49.4)	23 (41.1)	0.25

**Table 2:** Odds ratio of developing preeclampsia

Patient characteristics	Odds ratio	95% Confidence interval	
≥0.01 mg/dL decrease in serum creatinine	0.76	0.32	1.78
History of Preeclampsia	2.96	1.07	8.22
Chronic Hypertension	9.00	3.53	22.98
Diabetes mellitus			
Type 1	2.79	0.85	9.08
Type 2	4.78	1.63	14.02

## Discussion

### Principal findings

To our knowledge, the association between the physiologic decrease in serum creatinine in pregnancy and the development of preeclampsia has not been adequately studied. Our study demonstrates that the physiologic changes to the renal system caused by pregnancy and the associated decrease in serum creatinine is not protective from the development of preeclampsia in the face of other known risk factors. Interestingly, though type 1 diabetes mellitus, chronic kidney disease, and autoimmune disorders have been identified as important risk factors for preeclampsia [1], these were not associated with the development of preeclampsia in our patient population. This may be due to relatively low representation of patients with these conditions in our study population or this may reflect the efficacy of aspirin prophylaxis for lowering risk of preeclampsia in these high-risk patients. The finding that two-thirds of patients diagnosed with preeclampsia received aspirin therapy likely reflects the common implementation of low-dose aspirin therapy as a risk reduction strategy in high-risk patients rather than the ineffectiveness of low-dose aspirin. The fact that over three-fourths of patients who received low-dose aspirin did not develop preeclampsia emphasizes the benefits of this risk reduction strategy and the importance of continuing to identify additional risk factors and screening for potential candidates for aspirin prophylaxis early in their pregnancies.

### Clinical and research implications

Although obtaining a baseline serum creatinine is standard of care in patients with risk factors for preeclampsia early in pregnancy, it is otherwise not routine in patients at low risk for preeclampsia. Therefore, patients included in this study had a baseline creatinine measurement for either a specific diagnosis or workup, and thus may not be representative of the general population. Preeclampsia remains one of the major complications responsible for maternal mortality globally [8], and before eliminating a lack of decrease in baseline serum creatinine as a risk factor for preeclampsia based on our study findings, further studies should be completed, including prospective assessment of a diverse population.

A stronger conclusion can be drawn from a prospective study where serum creatinine may be trended several times between the first and second trimester to ensure a decrease in serum baseline creatinine was not missed due to the paucity of laboratory values. In addition, this prospective study design would likely capture a more accurate representation of the general population, as study subjects are not identified based on previously existing laboratory values.

### Strengths and limitations

Our study has several limitations, including those inherent in a retrospective cohort study. As mentioned earlier, a physiologic decrease in serum creatinine due to pregnancy can be noted as early as the sixth week of gestation. Our definition of baseline creatinine is based on this finding; however, it also relies on accurate dating, which was not extensively assessed in this study. With underestimation of dating, a creatinine believed to be drawn at 5 weeks may have been drawn at 7 weeks of gestation, for example. It is possible that, though a decrease in serum creatinine from baseline is not observed in this scenario, it may have already occurred prior to the considered baseline value. Furthermore, *Harel *et al*.* trended serum creatinine levels before, during and after pregnancy and noted a nadir in measurements between 16 and 20 weeks of gestation [8]. Measurements after 20 weeks were not considered in our study to control for the possibility of confounding factors that may impact serum creatinine values such as preeclampsia. Thus, a decrease in serum creatinine may not have been observed based on the timing of our laboratory values. In addition, our patient population may not be representative of the general population due to the inherent nature of which patients were most likely to have serum creatinine data available for inclusion. As such, we likely capture a higher risk cohort with respect to renal disease and preeclampsia development. This is consistent with the high rate of preeclampsia (14.3%) in our study compared to the estimated background rate of 2–8% [6].

## Conclusions

After controlling for risk factors associated with preeclampsia, this retrospective study showed that a decrease in serum creatinine from baseline was not associated with a decreased odds of developing preeclampsia in our patient population. Further studies are needed to determine if an absence in renal adaptations to pregnancy, including a decrease in serum creatinine, is associated with an increased odds of developing preeclampsia.

## Data Availability

For original data, please contact matt.grace@vumc.org.
